# Wearable Sensors for Precise Exercise Monitoring and Analysis

**DOI:** 10.3390/bios15110734

**Published:** 2025-11-03

**Authors:** Bo Su, Fengyu Li, Bingtian Su

**Affiliations:** 1 School of Physical Education, Guangdong Provincial Key Laboratory of Speed Capability Research, Su Bingtian Center for Speed Research and Training, College of Chemistry and Materials Science, Jinan University, Guangzhou 510632, China; 2 College of Chemistry, Zhengzhou University, Zhengzhou 450001, China

**Keywords:** wearable sensors, exercise monitoring, data precision, sports training

## Abstract

The adoption of wearable sensors for precision training has accelerated in recent years, yet most studies and reviews remain device- or feasibility-centric and lack a field-ready decision framework. This review organizes wearable sensing across four monitoring dimensions—physiological, kinematic, biochemical, and dynamic—and maps them onto three training pillars: physical, technical, and tactical. From the perspectives of athletes and coaches, we operationalize quality control, threshold, and feedback loop to translate measurement into action. We critically appraise key limitations, including signal robustness under high-intensity motion, inter-individual variability and limited model generalizability, cross-device data fusion and latency, battery life and wearability, privacy and data ownership, and limited accessibility beyond elite settings. Looking ahead, we advocate a shift from mere multidimensional measurement to a verifiable, reusable, and deployable precision-training ecosystem that delivers actionable metrics and clear decision support for practitioners.

## 1. Introduction

Accurate multidimensional data acquisition during physical activity is essential for optimizing training, preventing injuries, and enhancing performance. Continuous, real-time monitoring underpins individualized interventions, load evaluation, and early risk detection, aligning with the needs of precision training and rehabilitation [1,2,3]. Recent advances in muscle biology emphasize the plasticity of exercise metabolism and sex-specific adaptations, strengthening the biological rationale for data-driven monitoring [2,3].

Conventional approaches face common limitations: reliance on subjective ratings reduces reliability; laboratory or intermittent tests lack ecologically valid real-time measurements; and single indicators or modalities struggle to capture multiple parameters simultaneously and link them to training-competition contexts, thereby creating a measurement-to-decision bottleneck [4,5].

Wearable sensors help address these issues. Multimodal platforms, including IMUs, EMG, ECG, and sweat and other biofluid biochemical sensing, combined with low-power wireless and analytics, can continuously track internal and external loads with near-real-time feedback, providing a unified data foundation for mechanistic insight and practical optimization [6].

**Figure 1:**
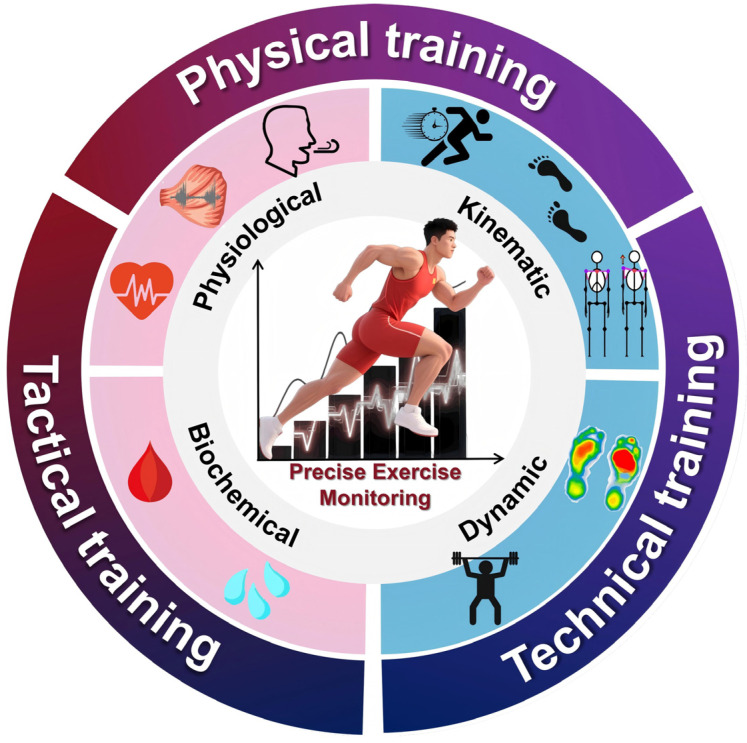
A diagrammatic overview of the review. The signals commonly used in exercise monitoring and their application scenarios, including physiological signals, biochemical signals, kinematic signals, and dynamic signals. The monitoring of these signals is often applied in physical training, technical training, and tactical training.

In the current wearable literature and reviews, a substantial proportion still prioritizes feasibility of signal acquisition and validity/reliability testing from device- or system-design perspectives, whereas decision workflows tailored to athletes/coaches remain underdeveloped [7,8,9,10,11]. Meanwhile, recent consensus statements and systematic reviews on training-load management-injury/illness risk and tactical/team spatiotemporal metrics emphasize internal and external load integration and contextualized application, moving wearable data from “signal-available” to “decision-ready” [1,12,13,14]. Accordingly, this review first structures the field into four dimensions: physiological, kinematic, biochemical, and kinetic; it then maps metrics onto three decision lines, conditioning, technical skills, and tactical coordination (as illustrated in Figure 1), and proposes a reusable framework of metric mapping, threshold setting, and feedback implementation to fill the device/validity-centric gap [1,6,12,13,14].

## 2. The Functions of Wearable Sensors in Exercise Monitoring

### 2.1. Physiological Monitoring

Wearable sensors facilitate real-time monitoring of the athletes’ physiological parameters, providing an efficient and non-invasive approach to evaluating internal physiological responses across different stages of exercise [15,16]. These devices are capable of continuously tracking dynamic changes in key physiological indicators, thereby providing valuable insights into the athlete’s performance status and physical condition. Wrist photoplethysmography (PPG) delivers generally accurate heart rate (HR) (but not energy-expenditure), with proper quality control (QC), such as strap fit, motion-artifact suppression, signal-to-noise ratio (SNR), and signal quality index (SQI),which supports intensity prescription and session monitoring [17]. Standardized heart-rate variability (HRV) reflects autonomic balance for individualized load/recovery [18,19]. Skin/ear temperature fusion aids core-temperature and heat-strain management; electrodermal activity (EDA) is maturing for field stress assessment [20,21,22,23].

The field of physiological monitoring faces the following challenges: (1) High-intensity motion, skin tone/perfusion, strap tension, and posture changes systematically degrade the stability of PPG and HRV; therefore, multi-wavelength/multi-path PPG, motion-adaptive filtering with SQI, fixed posture/time acquisition, and interpretation against a rolling individual baseline should be used to improve the repeatability of training-and-recovery decisions [17,18,19]. (2) Inferring core temperature from skin temperature and assessing stress via EDA are both context-dependent (ambient heat/humidity, sweating and blood-flow redistribution, skin contact, and hydration); thus, multimodal fusion (ear/skin/HR/acceleration) with covariate logging of ambient conditions and contact impedance and scenario-specific validation of thresholds and alerts is required [20,21,22,23].

### 2.2. Kinematic Monitoring

Kinematic analysis focuses on the temporal and spatial characteristics of human movement, offering critical insights into the mechanical execution and coordination of motion patterns. Through quantifying variables such as velocity, acceleration, displacement, and joint angles, kinematic assessment serves as a foundation for evaluating movement quality, efficiency, and potential asymmetries.

Field IMU kinematics now show increasing concurrent validity and test–retest reliability versus optical systems for running/walking spatiotemporal metrics and joint angles [22,23,24]. The 2024 ISB recommendations standardize frames, alignment, and reporting, while alignment-free and dynamic-calibration methods reduce environmental and placement errors [25,26,27]. Tooling such as OpenSense and personalized musculoskeletal models is facilitating laboratory-to-field deployment [28].

The field of kinematic monitoring faces the following challenges: (1) Integration drift, soft-tissue artifact, and segment-frame misalignment still cap joint-angle accuracy; consequently, functional calibration, short-window integration with constrained sensor fusion, and alignment-free methods should be coupled with cross-task and cross-population validation to ensure generalization [22,23,24,25,26,27]. (2) Methodological heterogeneity, spanning event detection, joint definitions, and reporting conventions, limits comparability and reproducibility; therefore, studies should adopt ISB guidance, publish mappings from raw data to derived metrics and sensor placement protocols, and report uncertainty estimates [24,25,28,29,30,31].

### 2.3. Biochemical Monitoring

Although wearable biochemical monitoring remains at an early stage, preliminary applications in sport/exercise are promising. Most systems leverage sweat sensing for noninvasive, continuous readouts of internal load and homeostasis, integrated into garments, bands, or patches with minimal motion interference [32,33,34,35]. Recent reviews highlight that coupling flexible microfluidics with electrochemical transduction is improving sensitivity and spatiotemporal resolution [32,36].

Current capabilities and analytes beyond lactate and electrolytes (Na^+^, K^+^), cortisol (stress), and glucose (energy metabolism) are emerging targets [34,35,37,38,39,40]. Evidence suggests stronger serum–sweat agreement for cortisol in several studies and reviews, whereas sweat–blood lactate correspondence is conditional and often inconsistent across sweat rates and exercise phases. In parallel, continuous glucose monitoring (CGM) of interstitial fluid is gaining traction in athletes, but benefits and best practices for non-diabetic users are still being defined [37,38,39,40,41,42,43].

The field of biochemical monitoring faces the following challenges: (1) Calibration and stability under dynamics. Exercise induces temperature/humidity swings, motion, and strain that drive baseline drift and noise. Electrochemical readouts are sensitive to reference stability, redox potential, pH, and temperature. Consensus recommendations include in vivo and near-body multi-point calibration, redox buffers, stabilizing conducting polymers, and concurrent temperature and flow sensing to improve reproducibility [32,44]. (2) Inter- and intra-individual and site variability in sweat rate and chemistry. Intensity, environment, heat acclimation, sex, and body site affect sweat rate and [Na^+^], causing mixing of new and old sweat and local dilution/concentration. Best practice is to report real-time sweat rate, sampling site, and conditions and to use one-way microfluidics and flow metering to mitigate mixing artifacts [32,36,45,46,47,48]. (3) Sweat–blood mapping and lag vary depending on the analyte. Transport mechanisms differ by analyte: ions (Na^+^, Cl^−^) and tightly regulated species (e.g., K^+^) generally do not mirror plasma. Lactate correspondence is phase- and rate-dependent and often nonlinear; cortisol and other passively diffusing small molecules show more reliability between sweat and serum in several reports. Studies should include parallel blood/sweat (or interstitial) sampling, lag reporting, and calibration modeling [32,37,38,39,42,46].

### 2.4. Dynamics Monitoring

Dynamic monitoring focuses on force-related parameters in sports movements, providing essential biomechanical insights into how the body interacts with external surfaces and equipment during physical activity. By quantifying variables such as ground reaction forces (GRFs), joint loading patterns, and force application timing, dynamic monitoring plays a critical role in performance optimization, technique evaluation, and injury prevention.

In-shoe pressure-sensing systems quantify plantar pressure, partial GRFs, and contact events in the field and show generally good validity and reliability in walking and running, although peak vGRF may be underestimated in high-impact landings with notable device- and task-specific differences [49,50,51]. In endurance and strength contexts, LPT/LVT and IMU devices deliver actionable KPIs for velocity-based training with strong reliability under exercise- and load-specific constraints [52,53]. When force plates are unavailable, IMU-based models with machine learning (ML) can estimate GRFs and impulses; recent SSL approaches improve generalization and data efficiency and are beginning to integrate PINN-style priors [54].

The field of kinematic monitoring faces the following challenges: (1) Robust mapping from pressure to force is particularly challenging in high-impact scenarios (jumping and landing) because insoles primarily capture normal pressure (not shear) and may saturate or underestimate peaks; therefore, individualized calibration, task-specific models, and high-rate event detection are needed to improve peak and loading-rate estimates, alongside explicit definition of usage [49,50,51]. (2) Mechanical outputs are highly sensitive to surface, footwear, and fatigue, and current models often lack transparency; consequently, validation should span surfaces/footwear/athletes with uncertainty reporting, and IMU-based ML or SSL approaches ought to disclose training-data distributions and synchronization references while leveraging PINN/physics priors to curb overfitting and enhance transferability [54].

## 3. Wearable Sensors Facilitate the Precision Sports Training Systems

The multidimensional data captured in Section 2 (physiological, kinematic, biochemical, and dynamic) are integrated here to enable precision across three pillars of athletic preparation: physical, technical, and tactical training. We explicitly link each sensor stream to decision rules (zones, thresholds, and feedback) and appraise technology readiness levels (TRLs) and remaining barriers to adoption (as illustrated in Table 1).

We cautiously adapt TRLs to use-case units (“sensor × application”) in sport and compute an indicative 1–9 score using five dimensions with gate caps and a conservative synthesis:

Dimensions (A–E): A validity/reliability (agreement vs. reference, test–retest, field replication); B robustness/transfer (across speed/environment/skin tone/placement; failure modes and SQI); C standards/guidelines (e.g., ISB, FIFA EPTS, third-party testing); D workflow and closed-loop (QC–threshold–feedback, latency, minimal KPI set); E scale and compliance (multicenter deployment, quality/regulatory alignment).

Gate caps: no field reference—TRL ≤ 5; lack of standards/certification/guidance ⇒ TRL ≤ 7; no actionable thresholds or real-time feedback —TRL ≤ 6.

Synthesis: weighted average (A, B, D = 0.25; C, E = 0.125), floor to the lower bound and apply gates; report uncertainty ranges.

We anchor this approach in ISO-16290 while aligning with digital-health DiMe V3/V3+ and NICE ESF evidence expectations; FIFA EPTS performance testing serves as an external benchmark for high-TRL tracking use-casess [55,56,57,58,59].

### 3.1. Physical Training

Physical fitness training within sports conditioning constitutes a process centered on systematic load stimulation. Its primary objective is to specifically enhance the athletes’ physical attributes, namely, strength, endurance, speed, agility, and flexibility, through the scientific modulation of training intensity, volume, frequency, and recovery periods. Its core goal is to induce adaptive physiological and biomechanical remodeling of the body to optimize specific competitive performance and enhance exercise tolerance. During implementation, training plans must be dynamically adjusted according to the athletes’ individual characteristics, specific requirements, and training phases, adhering to the principles of individualization and periodization.

Physical conditioning is driven by systematic modulation of intensity, volume, frequency, and recovery. Wrist photoplethysmography (PPG) provides generally accurate heart rate (HR) for zone-based prescription, whereas energy-expenditure estimates are unreliable at higher intensities. Standardized heart-rate variability (HRV) reflects autonomic balance and recovery status, and inertial measurement units (IMUs) provide spatiotemporal gait and running metrics (cadence, stride length, contact time) that flag fatigue-related technique drift and asymmetry. Fusing HR/HRV with IMU outputs constrains training zones more robustly, minimizing cumulative injury risk while improving adaptation specificity [17,18,19,39,42,60,61,62,63].

Using an ISO/NASA 1–9 TRL frame: PPG-HR is high-TRL (≈8–9) with multi-brand field validation; HRV in sport is mid-to-high TRL (≈6–7) but hinges on strict standardization (posture, time-of-day, breathing, sleep) to ensure repeatability; IMU spatiotemporal running metrics are high-TRL (≈7–8) with solid concurrent validity and test–retest reliability; by contrast, sweat lactate for real-time load regulation remains prototypical (low TRL ≈ 3–4) due to sweat-rate variability and uncertain sweat–blood mapping, so it should currently augment—not drive—training decisions. Key adoption barriers include motion/phenotype effects on PPG/HRV, IMU placement/alignment consistency across sessions, and context-specific calibration/thresholding for sweat analytics [17,18,19,39,42,60,61,62,63].

### 3.2. Technical Training

Technical training in sports constitutes a systematic process aimed at optimizing the accuracy, consistency, and biomechanical efficiency of movements through quantitative analysis and targeted correction of critical components within specific sport skills (e.g., kinematic sequences, force transmission pathways, and temporal coordination patterns). Its primary objective is to establish movement patterns that conform to the mechanical principles governing the human kinetic chain. In practice, training plans need to be dynamically adjusted in combination with athletes’ individual movement characteristics and specific technical standards.

Technical training aims to optimize accuracy, consistency, and mechanical efficiency via quantitative analysis and targeted correction. IMUs provide joint/segment timing and angles; in-shoe pressure systems deliver plantar-pressure maps and contact events as surrogates for ground reaction forces (GRFs); together they enable rapid, field-based feedback for high-speed, highly coordinated skills. Evidence from judged and cyclic sports shows feasibility across take-off, flight, and landing phases with actionable feedback loops [24,64,65,66,67,68,69,70,71,72,73].

A pelvis and bilateral thigh IMU configuration (sagittal angular kinematics) combined with bilateral in-shoe pressure sensors (contact onset, peak pressure, force–time surrogates) can quantify on-track: (i) reaction time (start signal synchronized to first plantar-pressure rise/hand release; audio trigger if instrumented blocks are unavailable); (ii) push-off and block-exit asymmetry (bilateral peak pressure and impulse ratios); and (iii) early-step spatiotemporal metrics (step length/time for the first two steps) and pelvic tilt, which relate to initial horizontal velocity. These metrics align with known determinants of start performance (propulsive block forces, first-stance contact time, angular-momentum control) and have been benchmarked against force plates/instrumented blocks or laser + IMU references, enabling concrete corrections such as shortening block contact, constraining excessive anterior pelvic tilt, reducing bilateral force asymmetry, and optimizing hip timing at touchdown [24,64,65,66,67,68,69,70,71,72,73].

IMU step-to-step kinematics in high-speed running show validated agreement with optical/laser/force-plate references (mid-to-high TRL ≈ 6–8). Pressure insoles yield reliable vGRF trends and contact events but may underestimate peak loads in high-impact phases (mid-to-high TRL ≈ 6–7). Adoption barriers include multi-device synchronization and time alignment, sampling-rate demands, context-specific pressure-to-force mapping, and integration into coaching workflows (real-time visualization, minimal KPI sets) [24,64,65,66,67,68,69,70,71,72,73].

### 3.3. Tactical Training

Tactical training in sports training refers to a training process that, based on specific competitive rules and opponent characteristics, systematically designs offensive and defensive strategies, positional coordination patterns, and dynamic response plans to optimize the decision-making efficiency and execution consistency of teams or individuals in competition scenarios. Its core goal is to establish a tactical cognition and behavioral coordination system that meets the needs of competitive situations. Through training that simulates strategy implementation, role collaboration, and situational transitions under competitive pressure, it improves the accuracy, adaptability of tactical execution, and the overall effectiveness of the team. In practice, training plans need to be dynamically adjusted in combination with opponent analysis, venue characteristics, and the individual tactical roles of athletes.

In team sports (e.g., football/soccer, basketball, rugby), GPS, LPS, and IMU plus HR form multi-level position, velocity, and load databases that quantify collective behaviors, namely, team centroid, surface area/convex hull, stretch index, and synchrony, across pressing, transitions, and compactness, while integrating external and internal load for contextualized decisions [14,50,74].

Wearable position/velocity tracking has standardized performance evaluation within FIFA’s Electronic Performance and Tracking Systems (EPTS) program and is high-TRL (≈8–9). However, collective tactical metrics show context-dependent reliability/sensitivity (sampling rate, algorithms, occlusion/indoor conditions), placing them mid-to-high TRL (≈6–7). Barriers include cross-system comparability, position errors under crowding/indoor conditions, and limited translation from metrics to coaching decision rules (e.g., thresholding for formation compactness or pressing triggers) [14,50,68,74].

**Table 1:** Mapping of sensor–application units to outputs, validation, standards alignment, gates, and indicative TRL.

Sens × Use-case	Primary Outputs	Validation Anchors (Refs)	V3/ESF Alignment	Gates Triggered	Indicative TRL (Range)	Notes
Wrist * PPG → HR zone prescription	HR, HR zones	ECG/chest-strap comparisons [17,60,61]	* V✓/A✓/C△	* —	8–9	Field-validated across sports; energy-expenditure (* EE) estimates not used for decisions.
* IMU (pelvis/thigh)→ sprint start profiling	Step time/length, pelvic tilt	Force-plate/laser + IMU [24,64,65,66]	* V✓/A✓/C△	* —	6–8	Requires high sampling and precise synchronization; align sensor frames consistently.
In-shoe pressure → * vGRF trend and contacts	Plantar pressure, contact events	Force-plate comparisons [50]	* V✓/A✓/C△	Peak underestimation → cap	6–7	Peaks may be underestimated in high-impact tasks; task-/shoe-specific calibration recommended.
Sweat lactate → real-time load regulation	[Lac], sweat rate	Sweat–blood mapping studies [39,62]	* V△/A△/C△	No actionable thresholds → cap	3–4	High inter-/intra-subject variability; use as adjunct signal, not primary driver.
CGM (interstitial) → fueling/pacing support	Glucose dynamics	Athlete reviews/pilot studies [42]	* V✓/A✓/C△	* —	5–6	Cautious use in non-diabetic athletes; interpret with nutrition/medical oversight.
GPS/LPS/* IMU (tactical) → formation compactness	Position, velocity; centroid/surface/stretch index	FIFA EPTS; positional-data reviews [14,68,74]	* V✓/A✓/C△	Collective metric sensitivity/context dependence → cap	8–9 (tracking); 6–7 (collective metrics)	Cross-system comparability issues; indoor/occlusion errors may increase.

* Abbreviations: PPG, photoplethysmography; IMU, inertial measurement unit; vGRF, vertical ground reaction force. V/A/C, DiMe V3 layers (Verification/Analytical/Clinical); “✓” indicates being basically in place; “△” indicates insufficient evidence or being contextualized ESF, NICE Evidence Standards Framework; EE, energy expenditure. “—“ indicates “not applicable”. Notes: Gate caps applied: no field reference—TRL ≤ 5; lack of standards/guidance—TRL ≤ 7; no actionable thresholds/real-time feedback—TRL ≤ 6.

## 4. Limitations and Practical Barriers

### 4.1. Sensor Accuracy and Robustness in Intense, Dynamic Movement (Signal Noise and Motion Artifacts)

**Figure 2:**
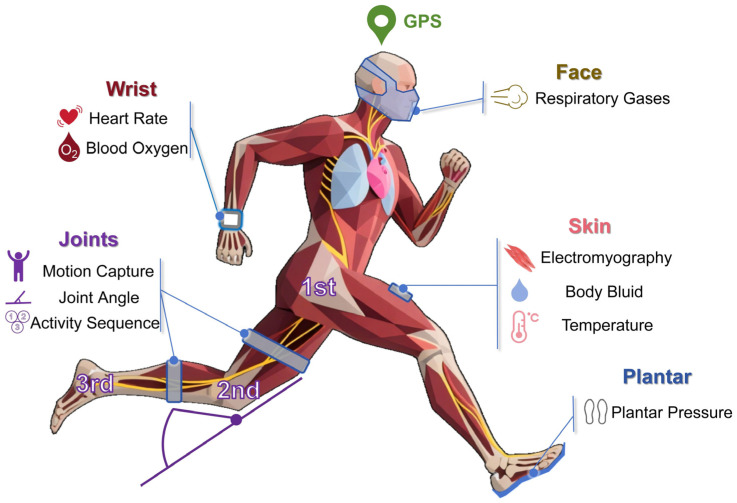
Common body parts and metrics for training monitoring. The rapid development of related technologies has enabled wearable sensors to find numerous applications in fields such as healthcare, energy, and the environment. However, the types of sensors used in the training monitoring field are relatively limited. Due to the complexity of sports training movements, wearable sensors must minimize their impact on athletes as much as possible.

During intense, dynamic movement, optical and inertial signals are vulnerable to motion artifacts, soft-tissue oscillations, and sensor micro-slippage. Wrist-PPG HR errors grow with vigorous arm swing and at high intensities; energy-expenditure estimates are particularly unreliable across brands [17,18,60]. Lower-limb IMU step-to-step metrics generally agree with optical/laser/force-plate references but are sensitive to placement/alignment, magnetic disturbance, and sampling-rate limits [64,68]. In-shoe pressure systems capture contact events and vGRF trends yet may underestimate peaks during high-impact tasks, requiring task- and shoe-specific calibration [50,62].Limited by the aforementioned factors, there are often few options for the placement of wearable devices (as illustrated in Figure 2).

### 4.2. Inter-Individual Variability and Model Generalization

Marked inter-individual and regional variability exists: HR/PPG depends on skin tone and perfusion [17,60]; HRV requires strict standardization and individual baselines to be interpretable [19,61]; sweat analytics are shaped by sweat rate and site, with unstable sweat–blood mapping that impedes threshold transfer [24,65,66]. Hence, one-size-fits-all models are brittle; personalized or hierarchical modeling with multi-site datasets is needed.

### 4.3. Data Fusion and Threshold Transfer (Bridging Measurement to Decision)

Multi-sensor fusion often degrades in the wild due to latency, synchronization, packet loss, and cross-system algorithmic differences, with collective tactical metrics particularly sensitive to sampling-rate and context (indoor/occlusion/crowding) [14,68]. Emerging biomarkers (e.g., sweat lactate) still lack actionable thresholds and on-field feedback workflows [65,66].

### 4.4. Energy Consumption, Battery Life, and Wearability

High-rate sensing and wireless streaming strain battery life; energy density and form factor limit 24/7 capture. Some epidermal/adhesive systems cause irritation, discomfort, or detachment, hurting compliance and continuity [6,75]. Energy optimization and harvesting are advancing, yet self-powered operation without sacrificing signal quality/rate remains challenging [6,75].

### 4.5. Data Security, Privacy, and Ownership of Athlete Biometrics

Athlete biometrics are sensitive personal data. Lawful processing requires compliance with GDPR and robust privacy-information management; in sport, charters such as the FIFPRO Player Data Rights emphasize transparency, consent, access/erasure, portability, and secondary-use limits [76,77]. Ownership and sharing across teams and vendors remain unsettled, posing ethical and compliance risks.

### 4.6. User Compliance, Comfort, and Accessibility (Cost and Equity)

Comfort, minimal interference, and esthetics determine long-term adherence [6,75]. Device/service/infrastructure costs constrain grassroots adoption; for some technologies (e.g., CGM in non-diabetic athletes), evidence and regulation are evolving, limiting access and equity [5,6,67].

## 5. Summary and Outlook

Biosensors and bioelectronic devices show substantial promise for exercise monitoring. Current systems integrate multidimensional biosignal sensors—such as electromyography (EMG), blood oxygen saturation (SpO_2_), heart rate variability (HRV), and lactate measurement—into wearable platforms to capture athletes’ physiological and kinematic status in real time. These platforms acquire comprehensive, high-resolution data reflecting internal and external training loads, providing an evidence base for designing and continuously optimizing personalized programs and for the early detection and prevention of sport-related injuries. By delivering real-time feedback on physiological thresholds and biomechanical parameters, athletes and coaches can implement evidence-based adjustments that improve training efficacy and safety.

**Figure 3:**
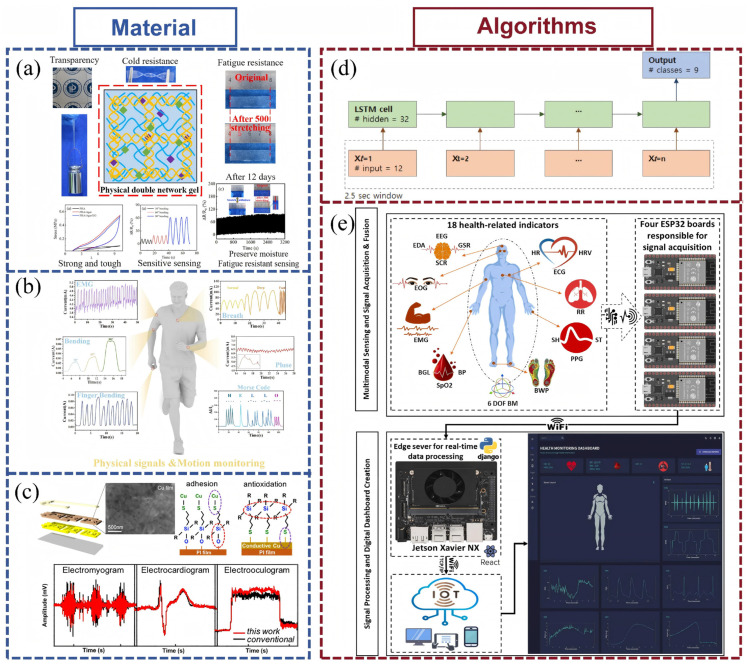
Advanced technologies for wearable sensors applied to exercise monitoring. (**a**) The PHA/Agar/EG hydrogel-based flexible sensor monitors different human motions and physiological activities [78]. (**b**) A multifunctional, integrated, flexible sensing platform based on biocompatible graphene/bacterial cellulose bioaerogel can be used for real-time monitoring of human health characteristic [79], and is reprinted (adapted) with permission from [19], Copyright 2023 Elsevier. (**c**) Nanophotonic-Sintered Copper Membrane with Ultrahigh Conductivity for Electrodes of Wearable Flexible Sensors [80]. (**d**) A “many-to-one” Long Short-Term Memory architecture for the classification of 9 types of human activities [81]. (**e**) An intelligent, IoT-enabled wearable multi-modal biosensing device [82] reprinted (adapted) with permission from [22], Copyright 2025 Elsevier.

In parallel, the adoption of advanced materials, such as flexible electronic skins, textile-integrated sensors, and smart sportswear, has markedly improved the comfort, stability, and noninvasiveness of data acquisition during dynamic activity. These innovations support prolonged wear and continuous monitoring under complex movement conditions without disrupting natural biomechanics. Consequently, long-term tracking of training adaptations, fatigue accumulation, and recovery progression has become more feasible and acceptable in both professional and recreational sport settings. Therefore, innovations in fields such as materials and algorithms can effectively drive the development of wearable sensors in the field of motion detection (as illustrated in Figure 3).

Looking forward, the field is poised to advance in precision, intelligence, and system integration. On the one hand, breakthroughs in materials science, especially pressure-sensitive materials [78], biodegradable substrates [79], and biocompatible conductive polymers [80], will enable further miniaturization, higher sensitivity, greater flexibility, and long-term skin compatibility. These gains can mitigate current limitations such as signal instability, motion artifacts, and adherence issues, supporting robust, unobtrusive data capture across varied training environments.

On the other hand, the deep integration of multimodal data fusion algorithms with artificial intelligence (AI) and machine learning (ML) frameworks will transform wearable systems from passive data collectors into active decision-support platforms [81,82]. By interpreting large-scale, heterogeneous biosignals, such systems can deliver real-time, personalized training recommendations, injury-risk estimates, and recovery guidance. Furthermore, establishing closed-loop systems that span data acquisition and preprocessing through adaptive feedback and automatic adjustment of training prescriptions will markedly improve the autonomy and responsiveness of digital training support platforms.

Materials and AI co-design will shift wearables from “measurable” to “actionable.” Conductive hydrogels provide a skin-sensor interface with high conductivity, strong adhesion, self-healing, and all-weather stability, preserving contact under large strain and cold to suppress motion artifacts. Coupled with lightweight on-device intelligence for context recognition, multimodal fusion, and personalized self-calibration, cleaner signals can be converted into low-latency, actionable training cues. This synergy targets long-term stability, comfort, and in-field calibration, accelerating deployment across the physical, technical, and tactical pillars [83,84].

This review contributes by mapping four wearable data domains, physiological, kinematic, biochemical, and dynamic, onto three training pillars: physical, technical, and tactical, thereby turning “what we can measure” into “what we can do,” quantified with use-case TRLs and operationalized through a quality control (QC), threshold, and feedback loop. The three pillars target load appropriateness (physical), movement economy (technical), and team coordination (tactical), aligning with core training objectives. For athletes, wearables should be light, stable, low latency, and provide concise cues anchored to personal baselines, with transparent privacy and data ownership; for coaches and teams, outputs should condense into on-screen thresholds and prescription suggestions with robust QC, cross-context reliability, and workflow compatibility. Future work should prioritize threshold-driven intervention trials, multi-site personalized and hierarchical modeling, and regularly updated use-case TRL assessments, advancing wearables from signal availability to true decision utility.

## Data Availability

No new data were created or analyzed in this study.
